# Enhanced Pathogenesis Caused by Influenza D Virus and Mycoplasma bovis Coinfection in Calves: a Disease Severity Linked with Overexpression of IFN-γ as a Key Player of the Enhanced Innate Immune Response in Lungs

**DOI:** 10.1128/spectrum.01690-21

**Published:** 2021-12-22

**Authors:** Adrien Lion, Aurélie Secula, Camille Rançon, Olivier Boulesteix, Anne Pinard, Alain Deslis, Sara Hägglund, Elias Salem, Hervé Cassard, Katarina Näslund, Maria Gaudino, Ana Moreno, Emiliana Brocchi, Maxence Delverdier, Siamak Zohari, Eric Baranowski, Jean-François Valarcher, Mariette F. Ducatez, Gilles Meyer

**Affiliations:** a IHAP, UMR1225, Université de Toulouse, INRAE, Ecole Vétérinaire de Toulouse, Toulouse, France; b INRAE, UE 1277, Experimental Infectiology Platform (PFIE), INRA-Val de Loire Research Centre, Nouzilly, France; c Swedish University of Agricultural Sciences, Host Pathogen Interaction Group, Department of Clinical Sciences, Uppsala, Sweden; d Istituto Zooprofilattico Sperimentale Della Lombardia e dell’Emilia Romagna Bruno Ubertini, Brescia, Italy; e Department of Virology, Immunobiology and Parasitology, National Veterinary Institute, Uppsala, Sweden; University of Prince Edward Island

**Keywords:** influenza D virus, *Mycoplasma bovis*, cattle, bovine respiratory disease, coinfection, immune response

## Abstract

Bovine respiratory disease (BRD) is a major disease of young cattle whose etiology lies in complex interactions between pathogens and environmental and host factors. Despite a high frequency of codetection of respiratory pathogens in BRD, data on the molecular mechanisms and pathogenesis associated with viral and bacterial interactions are still limited. In this study, we investigated the effects of a coinfection with influenza D virus (IDV) and Mycoplasma bovis in cattle. Naive calves were infected by aerosol with a French IDV strain and an M. bovis strain. The combined infection shortened the incubation period, worsened the disease, and led to more severe macroscopic and microscopic lesions compared to these parameters in calves infected with only one pathogen. In addition, IDV promoted colonization of the lower respiratory tract (LRT) by M. bovis and increased white cell recruitment to the airway lumen. The transcriptomic analysis highlighted an upregulation of immune genes in the lungs of coinfected calves. The gamma interferon (IFN-γ) gene was shown to be the gene most statistically overexpressed after coinfection at 2 days postinfection (dpi) and at least until 7 dpi, which correlated with the high level of lymphocytes in the LRT. Downregulation of the PACE4 and TMPRSS2 endoprotease genes was also highlighted, being a possible reason for the faster clearance of IDV in the lungs of coinfected animals. Taken together, our coinfection model with two respiratory pathogens that when present alone induce moderate clinical signs of disease was shown to increase the severity of the disease in young cattle and a strong transcriptomic innate immune response in the LRT, especially for IFN-γ.

**IMPORTANCE** Bovine respiratory disease (BRD) is among the most prevalent diseases in young cattle. BRD is due to complex interactions between viruses and/or bacteria, most of which have a moderate individual pathogenicity. In this study, we showed that coinfection with influenza D virus (IDV) and Mycoplasma bovis increased the severity of the respiratory disease in calves in comparison with IDV or M. bovis infection. IDV promoted M. bovis colonization of the lower respiratory tract and increased white cell recruitment to the airway lumen. The transcriptomic analysis highlighted an upregulation of immune genes in the lungs of coinfected calves. The *IFN-γ* gene in particular was highly overexpressed after coinfection, correlated with the disease severity, immune response, and white cell recruitment in the lungs. In conclusion, we showed that IDV facilitates coinfections within the BRD complex by modulating the local innate immune response, providing new insights into the mechanisms involved in severe respiratory diseases.

## INTRODUCTION

Bovine respiratory disease (BRD) is among the most prevalent pathological conditions in young cattle, resulting in poor animal welfare, significant economic losses, and increased antibiotic use in livestock (1). BRD is a multifactorial disease, resulting from complex interactions between pathogens and environmental and host factors, which can result in severe or fatal bronchopneumonia. Its complexity is often enhanced by the presence of mixed infections involving bacteria and viruses. The most important respiratory viruses involved in BRD are bovine respiratory syncytial virus (BRSV), bovine parainfluenza type 3 virus (BPIV-3), bovine viral diarrhea virus (BVDV), bovine coronavirus (BCoV), and sometimes bovine herpesvirus type 1 (BoHV1). Mannheimia haemolytica, Pasteurella multocida, Histophilus somni, and Mycoplasma bovis are the most common bacteria found and described in BRD (2). Among all pathogens, BRSV and M. haemolytica are considered major agents due to their ability to reproduce severe respiratory clinical signs when experimentally inoculated into naive calves (3, 4). In addition, recent metagenomics studies using next-generation sequencing (NGS) suggest a very high frequency of coinfections in BRD and have made it possible to identify new pathogens that are potentially involved (2, 5–9). These coinfections include well known respiratory pathogens but also viruses for which pathogenicity has not been demonstrated. For example, in North American BRD cases, coinfections frequently occurred between influenza D virus (IDV), BCoV, and bovine rhinitis A and B viruses (BRAV and BRBV, respectively) (5–7). Among these, IDV was isolated for the first time in 2011 in the United States from a swine exhibiting influenza-like syndrome (10). Despite its first detection in a pig, epidemiological studies highlighted a limited diffusion of IDV infection in this species. The high prevalence of IDV in cattle suggests this species as IDV’s main host (11–16). Alone, IDV induced only mild respiratory clinical signs in experimentally infected naive calves, despite replication in both the upper respiratory tract (URT) and lower respiratory tract (LRT) (17, 18). Metagenomics approaches suggested that IDV was implicated in BRD when associated with classical pathogens and/or with less known viruses, such as BRAV, BRBV, bovine nidovirus (BNV), bovine astrovirus (BAV), and bovine adenovirus type 3 (BAdV-3) (6–8).

Primary viral infections are well known to predispose the respiratory tract to secondary bacterial infections that lead to respiratory complications (19–21). Recent studies have shown that viral infection can promote bacterial superinfection but also that preexisting bacterial infections can promote viral shedding (20, 22). We recently identified frequent codetection of IDV and M. bovis in cattle with clinical respiratory signs on French veal farms (M. Gaudino, M.F. Ducattez, G. Meyer, personal communication) and Canadian dairy farms (23). Our field surveillance indicates that both IDV and M. bovis infections occurred at the same time, just after calves’ allocation, suggesting that M. bovis and IDV may act together as primary disease pathogens (Gaudino et al., personal communication). And yet, many cases of M. bovis are coinfections with other bacteria or viruses, as already suggested (24, 25).

To date, only one IDV and only one M. bovis experimental coinfection study have been performed in cattle. The first study suggested that primary IDV infection does not increase the clinical signs and susceptibility to secondary bacterial infection with M. haemolytica in calves (26). The second one explored the influence of BoHV-1 or BVDV type 2 primary infection on M. bovis superinfection in a calf model by modulating inoculation routes and doses (27). The authors showed that only calves first infected with BoHV-1 developed the respiratory disease after infection with M. bovis. As the association between M. bovis and IDV was observed in French veal calves and Canadian dairy cows with respiratory clinical signs, we assessed here whether this association of pathogens, inoculated at the same time, may worsen respiratory clinical signs and through what mechanisms. Our results confirmed that coinfection with IDV and M. bovis induces a more severe respiratory disease in calves than do monoinfections, with an increased innate immune response characterized by increased immune cell recruitment and a significant overexpression of gamma interferon (IFN-γ) mRNA in bronchoalveolar lavage (BAL) fluid samples.

## RESULTS

### Coinfection with IDV and M. bovis shortens the time to appearance of clinical signs and increases their severity.

**FIG 1:**
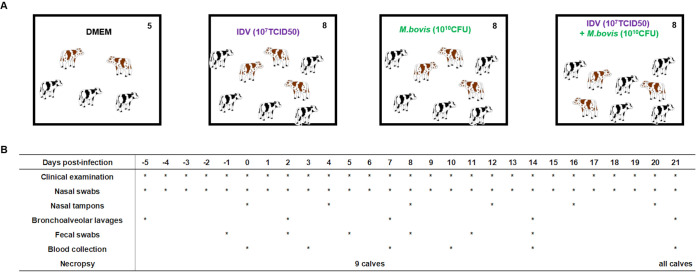
Experimental design. (A) Twenty-nine calves were isolated in four separate rooms. Eight calves were inoculated by nebulization with 10^7^ TCID_50_ of influenza virus D/bovine/France/5920/2014 or 10^10^ CFU of Mycoplasma bovis RM16 or combined pathogens. In the control group, five animals received DMEM medium. (B) Timeline and types of samples collected. *, day when sampling or clinical observation was performed.

Twenty-nine IDV- and M. bovis-negative calves were split into four groups in separate rooms. They were infected with IDV (*n* = 8), M. bovis (*n* = 8), or IDV plus M. bovis (*n* = 8) or not infected (control calves, *n* = 5) (Fig. 1). From the arrival of the calves to the end of the experiment, only minor clinical signs were observed for two calves (9713 and 9706) in the control calf group. These animals had a loss of appetite without respiratory signs. No other clinical signs (clinical score [CS] of <1, clinical cutoff of 1) were observed in the control group animals.

For the five calves that were inoculated with influenza virus D/bovine/France/5920/2014 and that were kept until 21 days postinfection (dpi), clinical signs started at 4.4 ± 1.1 days (mean ± standard deviation), with a peak at day 7.6 ± 1.8 and duration of elevated CS of 8 ± 1.2 days. As previously described (18), mild (four calves with a sum of daily CS [SCS] between 13 and 27) to moderate (one calf with an SCS of 44.7) respiratory clinical signs were observed in IDV-infected calves. The signs included mucus discharge, cough, slightly increased respiratory rate (35 to 40 breaths/min), and minor dyspnea with abnormal lung sounds for the most affected calf but without consequences on appetite, temperature, or general state. The five calves that were inoculated with M. bovis and that were kept until 21 dpi started showing clinical signs later, at 7.8 ± 1 days, with a peak at day 13.4 ± 1.5 and duration of CS of 9.6 ± 1.9 days. At the end of the experiment one calf (9642) was still sick. Clinical signs suggested infection of the URT, the trachea, and the primary bronchi. They were mainly characterized by a strong and frequent cough, fever, tachypnea and, to a lesser extent, mucus discharge, and slight dyspnea. Three calves developed mild disease (SCS values between 17 and 29), while the other two were more severely affected (SCS values of 35 and 44.7). Calves coinfected with IDV and M. bovis developed clinical signs similar to those observed in the M. bovis-infected calves except that the signs were more severe, with impairment of general condition for 3 calves, and started earlier than in the M. bovis group, at 5 ± 1.6 days, with a peak at day 8.6 ± 1.3 and duration of CS of 9.8 ± 2.5 days. Two calves had substantial clinical signs (SCS values of 39 and 58), with loss of appetite, general state impairment, decubitus, increased respiratory rate (30 to 60 breaths/min), mucopurulent discharge, numerous episodes of spontaneous cough, and abnormal lung sounds. For the other three calves, clinical signs were similar but less intense (SCS values of 20.5, 23.2, and 28.2). All animals had recovered by 21 dpi except one, which was still slightly ill.

**FIG 2:**
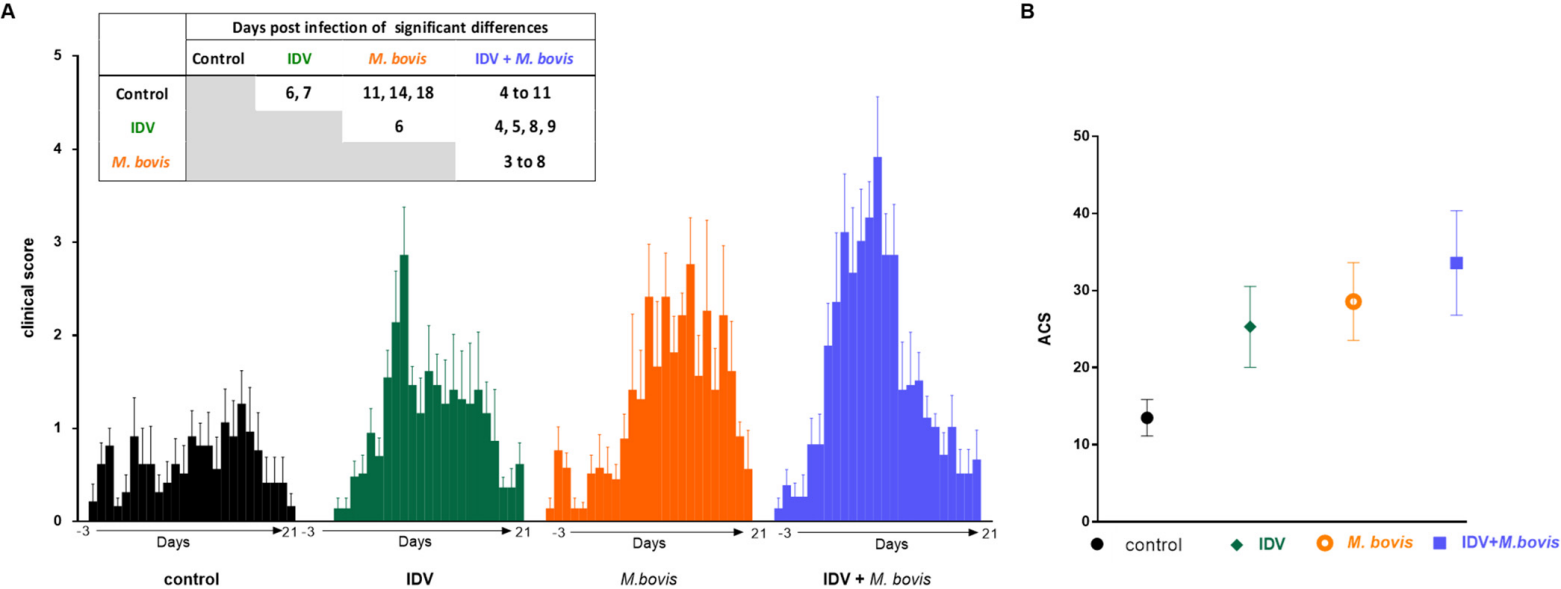
The coinfection increases the clinical scores. (A) Mean clinical scores with significant differences between groups. (B) Mean accumulated clinical score (ACS) for each group.

The average CS and the mean of the individual areas under the curves of daily clinical scores (ACS) with statistical differences are shown in Fig. 2. Significant differences in CS were detected between controls and coinfected (IDV+M. bovis-infected) calves from 4 to 11 dpi (0.0001 < *P* < 0.05) (Fig. 2A). The coinfected calves also showed statistical differences with the IDV-infected calves at 4, 5, 8, and 9 dpi but not at 6 and 7 dpi, which corresponds to the maximum of the CS of IDV-infected calves (statistical differences with the control calves). In addition, statistical differences were observed from 3 to 8 dpi between the coinfected and M. bovis-infected calves. Higher severity of disease in the coinfected calves was also detected when the mean areas under the curve (AUCs) of clinical scores were compared (Fig. 2B), although no statistical significance could be observed.

### Macroscopic and microscopic lesions were more severe in the coinfected calves than in IDV- or M. bovis-infected calves.

**FIG 3:**
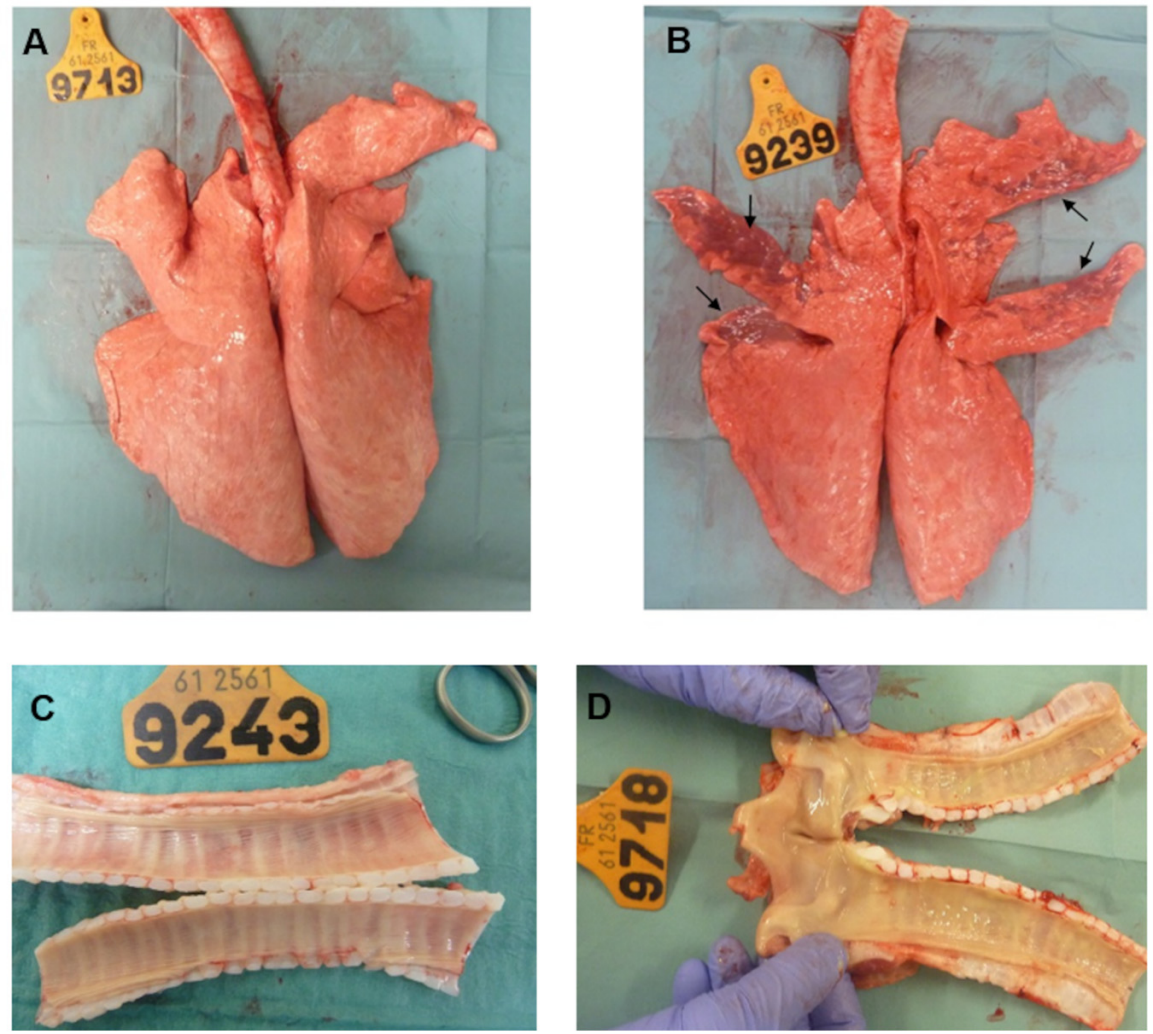
Types of macroscopic lesions observed. Macroscopic lesions in respiratory organs in infected compared to control animals. (A) Lung without macroscopic lesions of control calf 9713. (B) Thirty percent of the cranial and accessory lobes of the coinfected animal 9239 had atelectasis (arrows). (C) No gross lesions were observed at 6 dpi on the trachea of calf 9243 infected by M. bovis. (D) Tracheitis lesions with a fibrinopurulent exudate were observed on the mucosal surface of coinfected calf 9718 at 6 dpi.

To evaluate the gross lesions, animals were euthanized at 6 dpi (three animals per infected group) and 21 dpi (remaining calves). No gross lesions were found in control animals, either at 6 dpi or at 21 dpi (Fig. 3A). Similar to a previous study of experimental infection with IDV (18), only a single animal (9242) displayed minor macroscopic lung lesions of atelectasis and interstitial pneumonia at 6 dpi, covering 5 to 10% of the right cranial and accessory lobe surfaces (Fig. 3B). In the M. bovis-infected calves, no gross lesions were found at 6 dpi (Fig. 3C), and two calves (9697 and 9249) euthanized at 21 dpi had moderate lesions of nasal congestion and subacute interstitial bronchopneumonia, respectively. Remarkably, the main gross lesions were observed in the coinfected calves at 6 dpi. Two calves (9709 and 9718) had lesions of severe tracheitis with foci of necrosis and a fibrinopurulent exudate on the mucosal surface (Fig. 3D). The third calf (9239) had interstitial pneumonia with atelectasis of 30% of the cranial and accessory lobe surfaces. The latter calf did not harbor lesions on the trachea. At 21 dpi, three (9700, 9241, and 9244) and four (9700, 9705, 9241, and 9244) of five coinfected calves had lesions of tracheitis and acute interstitial pneumonia of minimal extent (5 to 10%), respectively. No other macroscopic lesions were observed in the other systems (digestive, nervous, urinary, etc.) of the 29 calves used in this experiment.

**FIG 4:**
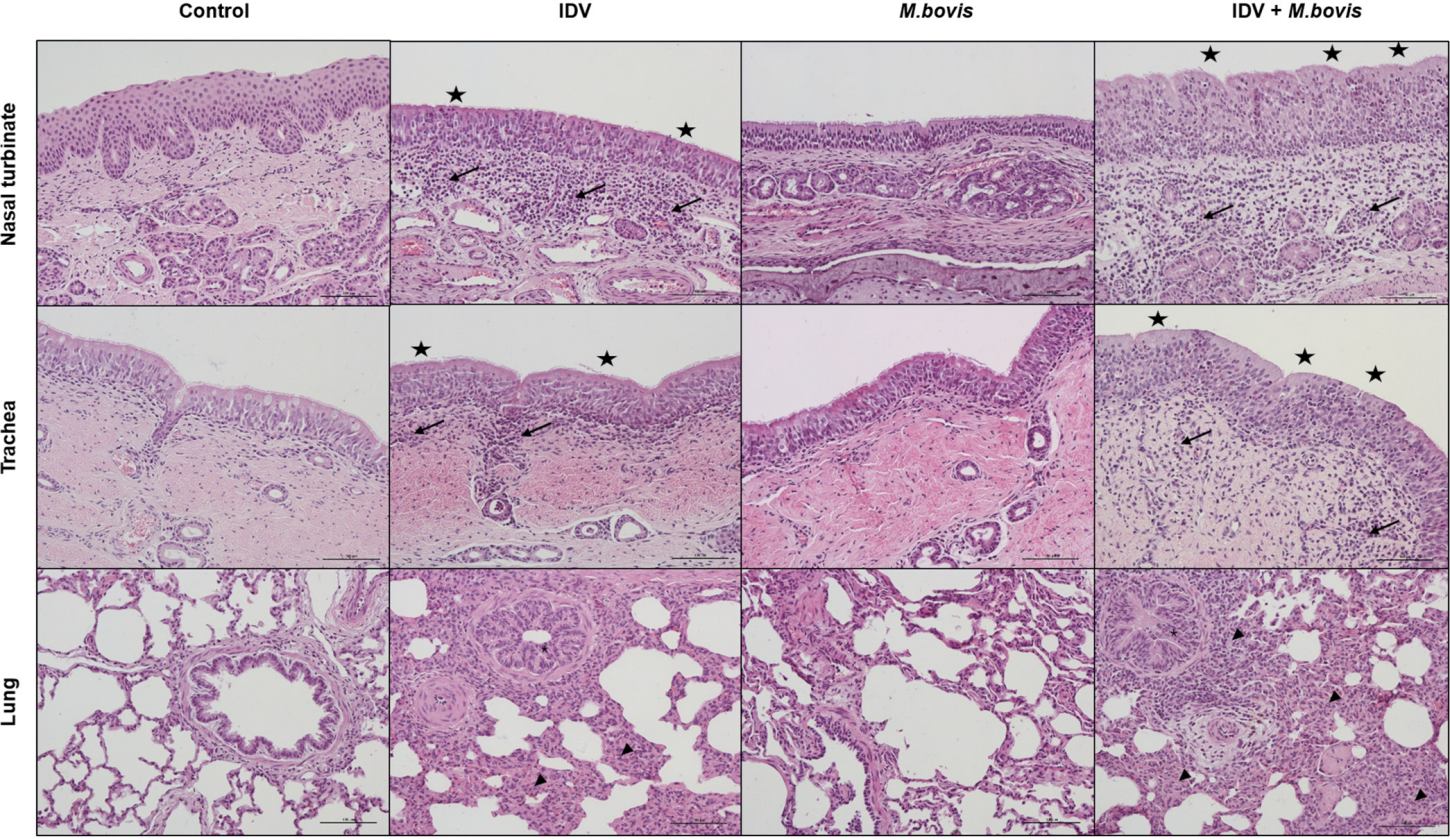
The coinfection induced greater lesions in the respiratory organs than did monoinfection. Hematoxylin and eosin staining of nasal turbinate (top), trachea (middle), and lung (bottom) tissue samples at 6 dpi. Magnification, ×200; scale bars, 100 μm. H&E-stained sections demonstrating a loss of ciliature and necrosis and exfoliation of the superficial mucosal epithelium (stars), an infiltration of the lamina propria by mononuclear cells (arrows), subacute bronchointerstitial pneumonia with neutrophils in bronchial lumens (asterisk), and neutrophilic and macrophagic alveolitis and peribronchial and septal lymphoplasmocytic infiltration (arrowheads).

**FIG 5:**
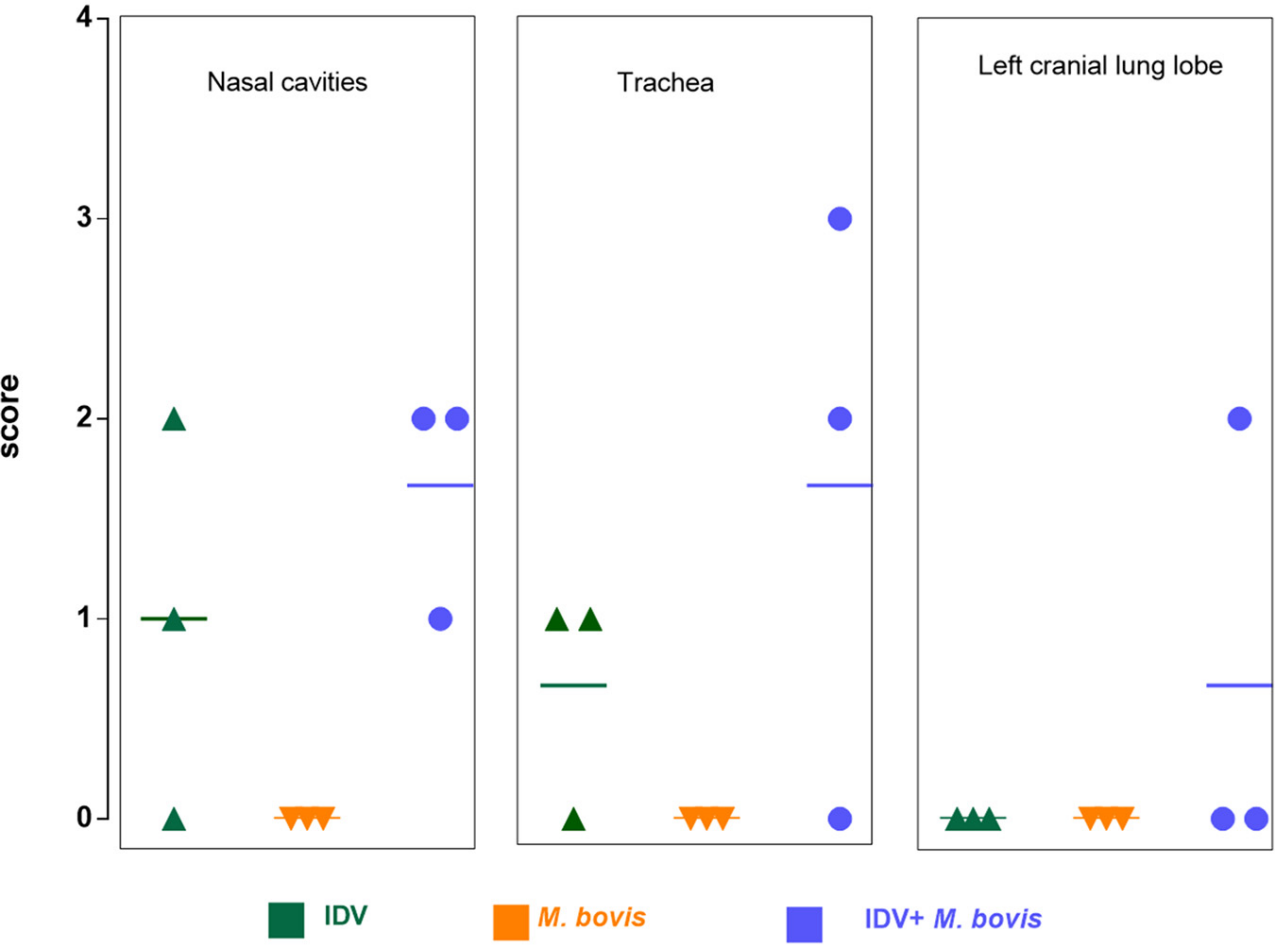
The coinfection increases the microscopic lesions. Mean and individual histologic lesion scores in nasal cavity, trachea, and lung for each infected group at 6 dpi.

At 6 dpi, microscopic lesions were only found in respiratory tissues (Fig. 4 and 5). The three IDV-infected animals had microscopic lesions in the nasal cavities and/or trachea, characterized by loss of cilia, necrosis and exfoliation of the superficial mucosal epithelium, and infiltration of the lamina propria by mononuclear cells (Fig. 4). All coinfected calves euthanized at 6 dpi showed lesions of rhinitis and tracheitis similar to those of IDV-infected calves, except that these lesions were more pronounced in the trachea (Fig. 5). Only coinfected calf 9709 had light microscopic lesions of subacute bronchointerstitial pneumonia in the left cranial lung lobe, characterized by neutrophils in the bronchial lumens, neutrophilic and macrophagic alveolitis, and peribronchial and septal lymphoplasmocytic infiltration in the lung. No microscopic lesions were found in animals infected by M. bovis and euthanized at 6 dpi. The macroscopic lesions observed at 21 dpi were not analyzed by histology.

### IDV and M. bovis coinfection increases white cell recruitment to the airway lumen.

To analyze the white cell response in the lung, the cellular compositions of BAL fluid samples at 2, 7, and 14 dpi were determined by using a cytospin system and May-Grünwald Giemsa (MGG) staining. Whole immune cells, macrophages, neutrophils, and lymphocytes from the different samples were counted (Table 1).

**TABLE 1:** Differential cell counts of total white cells, macrophages, neutrophils, and lymphocytes in BAL fluid samples from calves mono- or coinfected with IDV and M. bovis

Group (*n* = 5 calves/group)	Days postinoculation	Mean ± SEM (% unless otherwise indicated)			
		Total white cells (×10^6^ cells/ml)	Macrophages	Neutrophils	Lymphocytes
Uninfected control	2	5.6 ± 1.6	91.1 ± 1.4	4.8 ± 1.7	0.2 ± 0.2
IDV		5.4 ± 3.9	72.7 ± 9.9	12.6 ± 9.0	1.2 ± 1.0
M. bovis		8.6 ± 0.6	83.5 ± 11.1	8.3 ± 7.3	0.2 ± 0.1
IDV+M. bovis		6.9 ± 2.2	61.9 ± 8.8	23.4 ± 7.8	0.2 ± 0.1
Uninfected control	7	6.4 ± 3.0	76.6 ± 8.6	17.2 ± 7.8	0.5 ± 0.5
IDV		15.8 ± 8.0	58.9 ± 21.0	38.9 ± 21.0	0.6 ± 0.3
M. bovis		11.1 ± 4.6	51.8 ± 22.9	31.5 ± 14.3	0.5 ± 0.4
IDV+M. bovis		11.1 ± 5.5	50.7 ± 13.9	38.3 ± 14.6	1.0 ± 0.6
Uninfected control	14	6.0 ± 2.4	70.3 ± 4.3	4.4 ± 1.8	0.7 ± 0.5
IDV		13.6 ± 5.0	68.2 ± 10.8	7.3 ± 4.7	0.7 ± 0.6
M. bovis		11.9 ± 8.4	68.0 ± 9.8	13.2 ± 6.6	1.6 ± 0.6
IDV+M. bovis		4.4 ± 2.5	58.4 ± 17.2	26.2 ± 16.7	1.4 ± 1.4

In the control calves, the mean whole immune cell count (WCC) and neutrophil and macrophage counts remained stable during the study. However, a small progressive increase in the lymphocyte mean count was detected in control calves between 2 and 14 dpi (Table 1).

In the IDV-infected calves, the mean WCCs were higher at 7 and 14 dpi than at 2 dpi. This increase was mainly due to the maintenance of a high number of macrophages and an increase in neutrophil recruitment (26.3% increase between 2 and 7 dpi). The mean lymphocyte counts were also higher at 7 and 14 dpi than at 2 dpi. However, the percentage of lymphocytes over other white cell types was higher at 2 dpi than later during infection. Moreover, compared to the control calves, the IDV-infected calves tended to have a higher level of lymphocytes at 2 dpi (0.2% versus 1.2%) (Table 1).

In the M. bovis-infected calves, the WCCs tended to differ between 2 and 7 dpi or 2 and 14 dpi, mainly due to the increases in the neutrophil fraction, with a 23.2% and a 4.9% increase, respectively. The numbers of lymphocytes increased between 2 and 7 dpi and between 2 and 14 dpi, with 0.3% and 1.4% increases, respectively (Table 1).

In the IDV+M. bovis-infected calves, compared to the control calves, the mean WCC tended to be increased at 2 dpi mainly due to the high level of the neutrophil count (23.4%). The most significant change for the coinfected calves was observed at 7 dpi, when the WCC was increased by 1.6-fold compared to its level at 2 dpi, while it did not change in the controls between these two time points. The diapedesis of neutrophils into the lumen of the airways (14.9% increase in neutrophils in BAL fluid samples between 2 and 7 dpi) was mainly responsible for this increase of the WCC in the coinfected calves, while the number of macrophages was relatively stable between these two time points. The fraction of lymphocytes increased by 1.0% between 2 and 7 dpi. At 14 dpi, the WCC returned to the initial state in the coinfected calves; however, the neutrophil and lymphocyte fractions remained high, while the percentage of macrophages was reduced by 3.5% compared to the percentage at 2 dpi (Table 1).

Due to the individual variations, none of the differences in white cell-type counts were statistically significant.

### IDV infection promotes M. bovis colonization of the URT.

**FIG 6:**
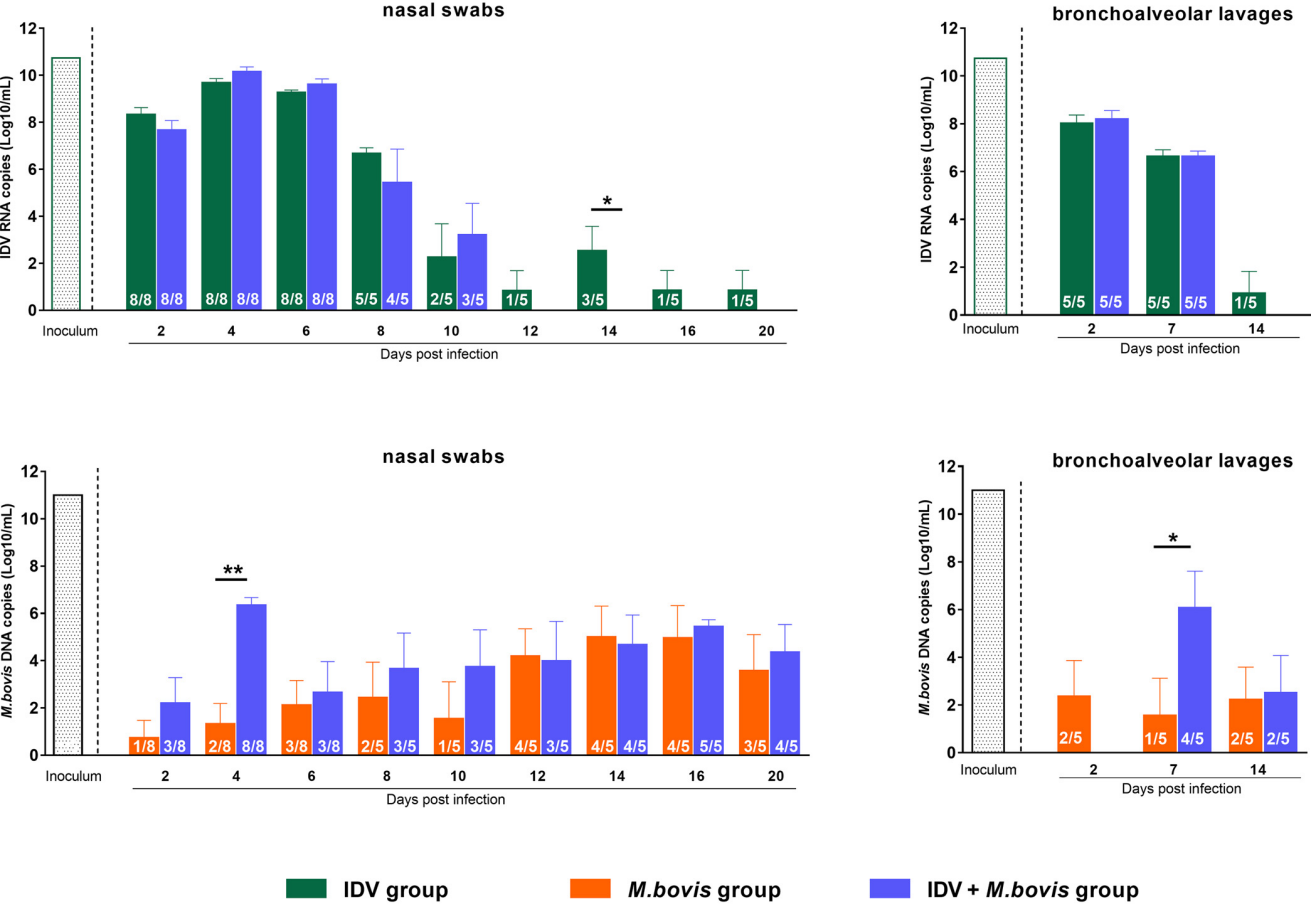
IDV infection promotes M. bovis colonization of the upper and lower respiratory tracts. Virus titers (top) and bacterial titers (bottom) in nasal swab (left) and bronchoalveolar lavage fluid (right) samples from calves at 0 to 20 dpi and 0 to 14 dpi, respectively. Data represent the mean values ± SEM of IDV RNA and M. bovis DNA copies (log_10_/ml) measured by RT-qPCR and qPCR, respectively. The number of positive animals/total number of animals is indicated in each bar. The titrations of the IDV (10.7 log_10_ RNA copies/ml) and M. bovis (11 log_10_ DNA copies/ml) inoculums were obtained on day zero just before they were administered to the calves by nebulization. *, *P* ≤ 0.05; **, *P* ≤ 0.01.

To assess the shedding of IDV in the URT, the presence of IDV RNA was monitored by reverse-transcriptase quantitative PCR (RT-qPCR) in deep nasal swab samples between 0 and 20 dpi (Fig. 6, top). No IDV was detected in samples from controls and M. bovis-infected calves. From 2 to 20 dpi, IDV was detected in IDV-infected calves, with a peak of 9.9 log_10_ RNA copies/ml at 4 dpi. In the IDV+M. bovis-infected calves, most animals were positive for IDV from 2 to 10 dpi, with a peak of 10.4 log_10_ RNA copies/ml at 4 dpi. All calves were positive for IDV from 2 to 8 dpi in the IDV group and from 2 to 6 dpi in the coinfected group (Fig. 6, top).

Mycoplasma bovis was not detected in the controls, as assessed by qPCR in nasal swabs collected between 0 and 20 dpi. The M. bovis replication kinetics from 2 to 20 dpi showed maximum values of 5 log_10_ (14 dpi) and 6.4 log_10_ (4 dpi) DNA copies/ml in M. bovis-infected and IDV+M. bovis-infected calves, respectively (Fig. 6, bottom). From 14 dpi, a large majority of calves (80%) were positive for M. bovis in M. bovis- and IDV+M. bovis-infected calves.

Finally, in the IDV+M. bovis-infected group, all animals (8/8) were double positives for IDV and M. bovis at 4 dpi in nasal cavities.

### IDV infection promotes M. bovis colonization of the LRT.

We then assessed the IDV and M. bovis replication in BAL fluid samples (Fig. 6) and trachea and lung specimens (Table 2). No IDV was detected in control and M. bovis-infected calves, and no M. bovis was found in control and IDV-infected animals.

**TABLE 2:** Virus and bacteria detection in trachea and lung of calves mono- or coinfected with IDV and M. bovis

Organ	Days postinfection	Mean copy number (log_10_/30 mg tissue) of RNA or DNA in indicated group (no. of positive animals/total number of animals)^*a*^:							
		IDV RNA				M. bovis DNA			
		Control	IDV	M. bovis	IDV+M. bovis	Control	IDV	M. bovis	IDV+M. bovis
Trachea	6		6.81 (3/3)	ND (0/3)	9.09 (3/3)_ABC_		ND (0/3)	ND (0/3)	4.79 (3/3)
	21	ND (0/5)	ND (0/5)	ND (0/5)	ND (0/5)	ND (0/5)	ND (0/5)	3.10 (4/5)	2.45 (2/5)
Lung	6		6.26 (3/3)	ND (0/3)	9.53 (3/3)		ND (0/3)	3.06 (2/3)	4.39 (2/3)
	21	ND (0/5)	4.32 (2/5)	ND (0/5)	ND (0/5)	ND (0/5)	ND (0/5)	ND (0/5)	3.88 (1/5)

a IDV RNA and M. bovis DNA copies were quantified by RT-qPCR and qPCR, respectively. ND, not detected. Statistical analyses were obtained with Bonferroni’s multiple-comparison test by comparing each group with a *P* value of <0.01. Significant differences between IDV+M. bovis-infected calves and the other groups are represented by letters as follows: a, uninfected controls; b, IDV-infected calves; and c, M. bovis-infected calves.

All IDV-infected and IDV+M. bovis-infected calves tested were positive for the virus in BAL fluid samples at 2 and 7 dpi (5/5) and in trachea and lungs at 6 dpi (3/3). In IDV-infected calves, the maximum viral titers were 8.5 (2 dpi), 6.8, and 6.3 (6 dpi) log_10_ RNA copies/ml in BAL fluid samples and trachea and lung specimens, respectively. Viral titers in coinfected animals were greater than in the IDV-infected calves, with maximum titers of 8.6 (2 dpi), 9.1, and 9.5 (6 dpi) log_10_ RNA copies/ml in BAL fluid samples and trachea and lung specimens, respectively. Unlike in the IDV-infected calves, no virus was detected in BAL fluid samples at 14 dpi or organs at 21 dpi in the IDV+M. bovis-infected calves.

Considering M. bovis, only 40% (2/5) of the BAL fluid samples from the M. bovis-infected calves were positive for the bacteria from 2 to 14 dpi, with a maximum of 2.4 log_10_ DNA copies/ml at 7 dpi. In this group, M. bovis was exclusively detected in two of three lung samples at 6 dpi and in 4 of 5 trachea samples at 21 dpi. In comparison, most positive samples (4/5) with higher titers of bacteria (average of 6.1 log_10_ copies/ml) were observed in BAL fluid samples of the IDV+M. bovis-infected calves at 7 dpi. The three coinfected animals euthanized at 6 dpi had at least 1 log_10_ M. bovis copies/ml more in the lung (*n* = 2/3) and trachea (*n* = 3/3) than the calves infected only with the bacteria.

The isolation of M. bovis was confirmed in the PCR-positive lung samples. In addition, all PCR-negative samples were also negative when isolation was attempted. These results confirm that IDV and M. bovis can infect the LRT of calves and that the combination of the two pathogens increases the M. bovis colonization of the lungs.

### Mono- and coinfections induce a humoral response against IDV in cattle.

**FIG 7:**
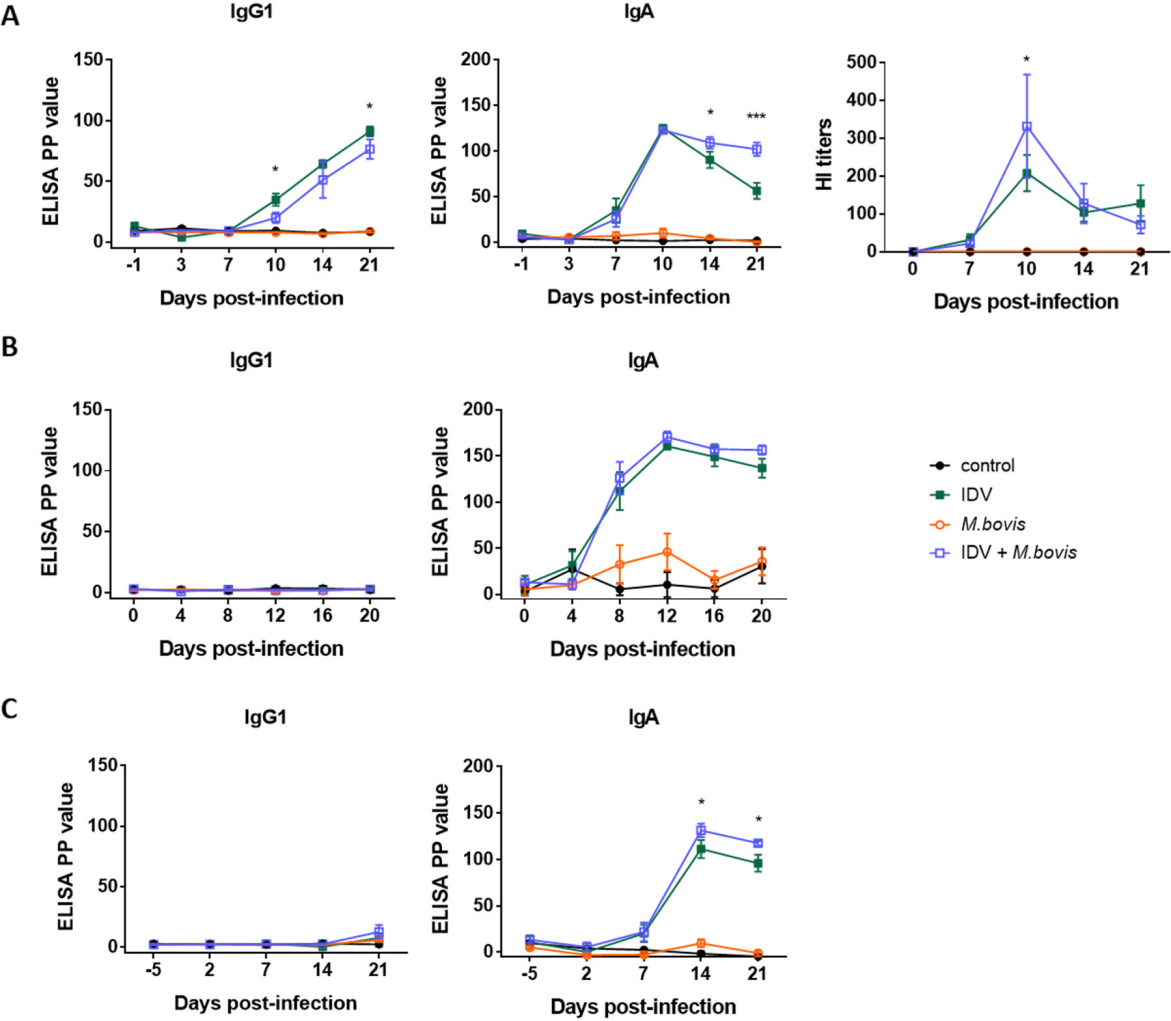
IDV induced a rapid host humoral response in mono- and coinfected calves. Total antibody, IgG1, and IgA titers from serum (A), nasal swab (B), and bronchoalveolar lavage fluid (C) samples from five calves per group were measured by HI assay with influenza virus D/bovine/France/5920/2014 for the IDV-specific antibodies and IDV-specific indirect ELISAs for IgG1 and IgA. Values are presented as means ± SEM of the percent positivity (PP). *, *P* ≤ 0.05 and ***, *P* ≤ 0.001 for statistically significant differences between IDV-infected and coinfected groups using Bonferroni’s multiple-comparison test.

All animals were seronegative for IDV and M. bovis before infections. To assess the humoral response, the antibodies against IDV from sera were first measured by hemagglutination inhibition (HI) assay with influenza virus D/bovine/France/5920/2014. Calves seroconverted against IDV from 7 dpi in both the IDV- and IDV+M. bovis-infected groups (Fig. 7A).

The presence of IDV-specific IgG1 and IgA was then measured in serum samples, deep nasal swab samples, and BAL fluid samples (5 calves per group) using enzyme-linked immunosorbent assay (ELISA) (Fig. 7). IDV-specific IgA and IgG1 were detected in sera of calves infected with IDV or IDV+M. bovis from 7 and 10 dpi, respectively (Fig. 7A). The levels of IDV-specific serum IgG1 were significantly higher in the IDV-infected calves than in the IDV+M. bovis-infected calves at 10 and 21 dpi, and the levels of IDV-specific serum IgA were higher in IDV+M. bovis-infected calves than in IDV-infected animals at 14 and 21 dpi. No IgG1 was detected in nasal swab samples (Fig. 7B) or BAL fluid samples (Fig. 7C) in IDV- and IDV+M. bovis-infected calves. Compared to the control calves, the mean levels of IDV-specific nasal IgA were higher in the IDV- and IDV*+*M. bovis-infected calves from 8 dpi, with maximal titers at 12 dpi. In BAL fluid samples, IDV-specific IgA was detected between 7 and 21 dpi, with maximal titers at 14 dpi, and the IgA titers were significantly higher in coinfected calves than in IDV-infected calves at 14 and 21 dpi.

These results demonstrate that the IDV infection induces a rapid local and systemic humoral immune response.

No anti-M. bovis antibodies were detected by ELISA in any samples, suggesting a delayed humoral response against *Mycoplasma* infection (data not shown).

### The IDV and M. bovis coinfection increases the innate immune response in BAL fluid samples.

**FIG 8:**
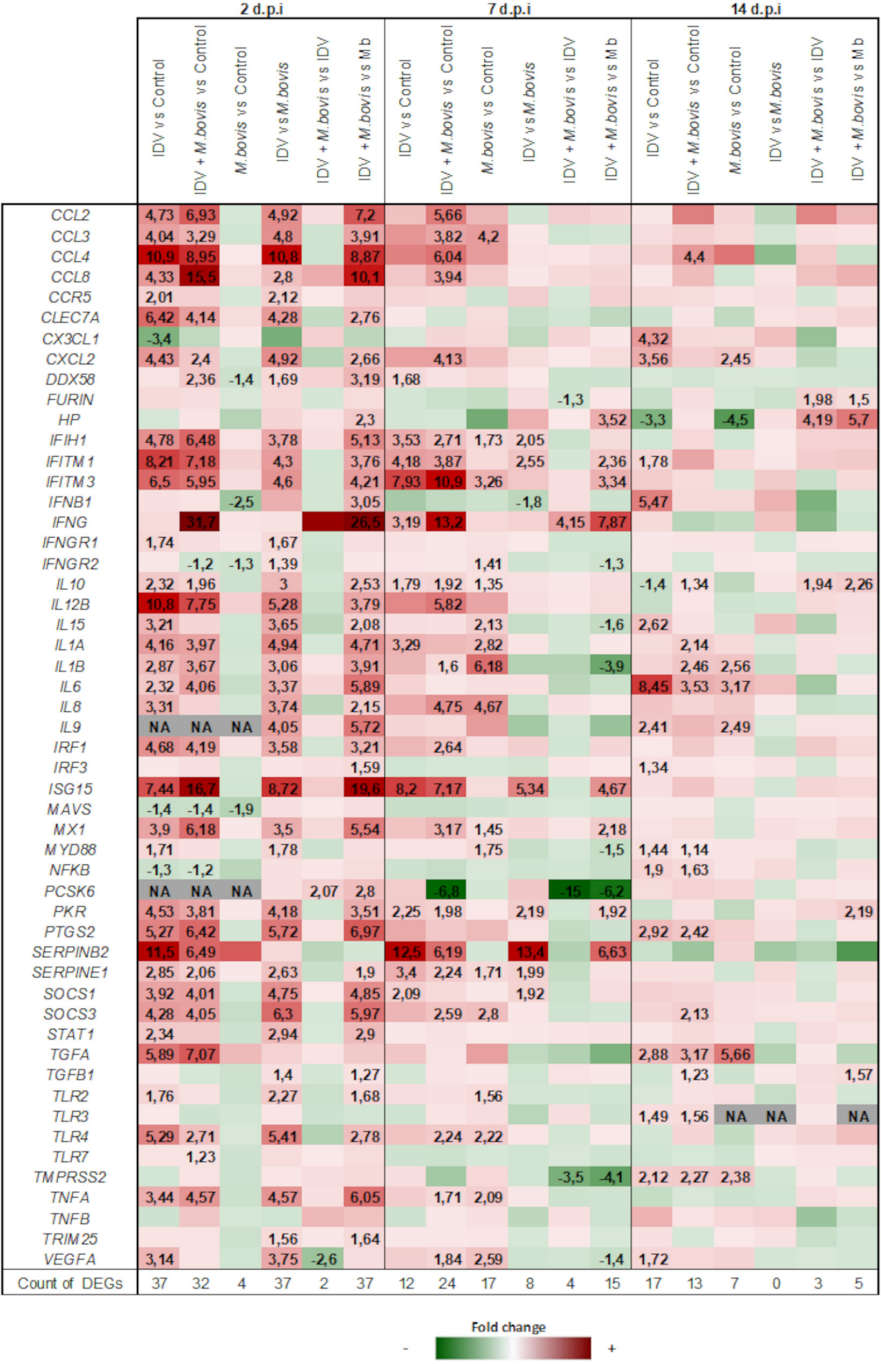
The coinfection increased the innate immune response in BAL fluid samples. Fold changes in the mRNA expression (Fluidigm) of 52 bovine genes from BAL fluid samples of mono- or coinfected calves. The fold changes in mRNA expression were calculated for pairs of groups at 2, 7, and 14 dpi. Positive fold changes are colored in red, and negative fold changes in green. The fold change values of differentially expressed genes (DEGs) with a significant difference (*P* < 0.05) are presented.

To explore the local host innate immune response, the expression levels of 52 targeted genes in BAL fluid samples were analyzed by Fluidigm PCR. These genes included those coding for pathogen recognition receptors (PRRs), cytokines, chemokines, antiviral molecules, cytokine signaling proteins, growth factors, and proteases (Table S1). No significantly different levels of expression between the four groups were observed 5 days before infection. The fold changes (FC) of gene expression levels and differentially expressed genes (DEGs) between groups at 2, 7, and 14 dpi are represented in Fig. 8.

IDV induced a quick immune response characterized by a large number of DEGs and fold changes in intensity at 2 dpi. In comparison to the gene expression in the controls, 37 of 52 genes were differentially expressed at 2 dpi, while 12 and 17 DEGs were identified at 7 and 14 dpi, respectively. Most of the genes (34/37) were overexpressed at 2 dpi. A strong antiviral response, characterized by the overexpression of IFN-stimulated genes (ISGs) (*IFITM1*, *IFITM3*, *ISG15*, *MX1*, and *PKR*) but a limited IFN-I response (*IFN-β1*) was identified. Moreover, the high levels of chemokines (*CCL2*, *CCL3*, *CCL4*, *CCL8*, *CXCL2*, and *IL-8*), interleukins (*IL-1α*, *IL-1β*, *IL-6*, *IL-10*, *IL-12β*, and *IL-15*), and *TNF-α* demonstrated the setting up of a proinflammatory state with a potential to recruit, activate and induce cellular proliferation of leukocytes in the lung. Lymphocyte activation was confirmed by the overexpression of *IFN-γ*, mainly at 7 dpi. The viral infection led to significant overexpression of PRR genes, mainly *DDX58*, *IFIH1*, *TLR2*, and *TLR4*. In addition, a limited increase of *TLR3* and *TLR7* was found at 2 dpi. Some signaling molecules (*IRF1*, *MAVS*, *MyD88*, *NF-κB*, *SOCS1*, *SOCS3*, and *STAT1*) and growth factors (*TGFα* and *VEGFA*) were also significantly dysregulated during IDV infection.

Considering M. bovis infection, we found a late and low local host response with a limited dysregulation of targeted genes. In comparison to the gene expression in the controls, only four genes (*DDX58*, *IFN-β1*, *IFNGR2*, and *MAVS*) were statistically underexpressed after 2 dpi with M. bovis. Most DEGs were identified at 7 dpi, with a total of 17 genes overexpressed. These genes play roles in the proinflammatory response and white cell recruitment (*CCL3*, *IL-1α*, *IL-1β*, *I-L8*, *IL-10*, *IL-15*, and *TNF-α*) and also in antiviral and signaling pathways (*IFIH1*, *IFITM3*, *IFNGR2*, *MX1*, *MyD88*, *SOCS3*, *TLR2*, and *TLR4*). The comparison between gene expression in IDV- and M. bovis-infected calves confirms the induction of a faster and stronger host response by IDV.

A rapid and high response was finally observed in the coinfected calves. In comparison to the gene expression in the controls, 32 DEGs were found, with 29 overexpressed and 3 underexpressed at 2 dpi. Compared to the gene expression in the controls, the coinfection led to 24 and 13 DEGs at 7 and 14 dpi, respectively. In addition to the DEGs found in the IDV-infected group, the coinfection induced significant overexpression of *CCL2*, *CCL3*, *CCL4*, *CCL8*, *CXLC2*, *IL-1β*, *IL-8*, *IL-12β*, *IRF1*, *MX1*, *SOCS3*, *TLR4*, *TNF-α*, and *VEGFA* and downregulation of *PCSK6* at 7 dpi. Nevertheless, compared to the gene expression in the IDV-infected calves, only the overexpression of *IFN-γ* and downregulation of *FURIN*, *PCSK6*, and *TMPRSS2* were statistically different in the coinfected versus the IDV monoinfected calves at 7 dpi. Many differences were identified between the coinfected and M. bovis-infected calves, particularly at 2 and 7 dpi. Finally, only the overexpression of *IFN-γ* and the downregulation of *PCSK6* and *TMPRSS2* were statistically different when the coinfected group was compared to the IDV- and M. bovis-monoinfected groups at 7 dpi (Fig. 8).

**FIG 9:**
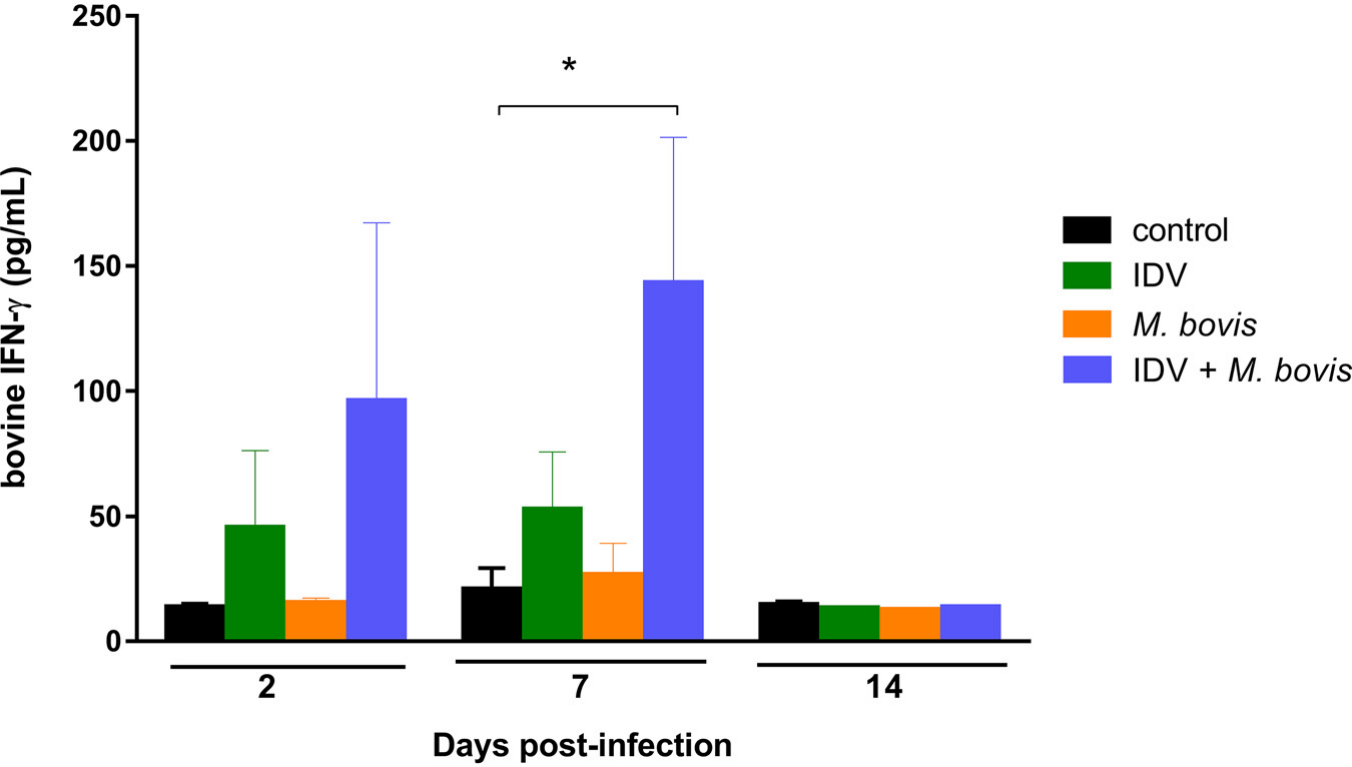
The coinfection increased the IFN-γ synthesis in BAL fluid samples. Bovine IFN-γ levels were measured in BAL fluid samples by sandwich ELISA. Data are presented as mean values ± SEM for the cytokine concentrations (pg/ml). *, *P* ≤ 0.05 for statistical difference between control and coinfected groups using Bonferroni’s multiple-comparison test.

To confirm the overexpression of *IFN-γ*, we analyzed the production of this cytokine by ELISA. We found increased production of the IFN-γ cytokine in BAL fluid samples of IDV-infected and coinfected groups at 2 and 7 dpi (Fig. 9). Compared to the IFN-γ production in the control group, the IFN-γ production of the IDV group was increased on average by 3.3- and 2.5-fold at 2 and 7 dpi, respectively. The highest overproduction was observed in the coinfected group, with 7.1- and 6.9-fold increases at 2 and 7 dpi, respectively. No real differences in cytokine production were measured between the control and the M. bovis-infected group. At 14 dpi, the IFN-γ protein levels were similar in all groups.

## DISCUSSION

In agreement with our previous study, where the same IDV challenge strain, inoculation dose, and route were used (18), and other similar studies (17, 26), we confirmed that IDV alone causes moderate respiratory clinical signs. Furthermore, the replication kinetics, the nasal, tracheal, and lung lesions, and the short IgG response were all similar to our previous results (18). We also used this experiment to further investigate the intestinal tropism of influenza virus D/bovine/France/5920/2014 previously described by Oliva et al. (28) in a mouse model. We were able to detect IDV by RT-qPCR in fecal swab samples (2/8 calves) at 5 dpi and in the jejunum (1/3 calves) at 6 dpi (data not shown), corresponding to the viral replication peak in the respiratory tract. Intestinal tropism and infectivity must be confirmed by immunohistochemistry and isolation studies, but these preliminary results raise the question of possible IDV contamination through the digestive tract, in addition to the aerosol transmission previously described (18).

In this study, we chose to do a coinfection at the same time because it better mimics the field conditions of BRD, in which veal calves from many herds are grouped for a short time, allowing rapid and concurrent contaminations by pathogens. Three- to 8-week-old calves were used, as in other studies where clinical signs and lesions typical of M. bovis pneumonia were successfully reproduced (29–32). Using the recently isolated French M. bovis strain RM16 and a challenge by nebulization, we were able to induce cough, increased nasal discharge, fever, and tachypnea, especially from 8 to 18 dpi. These observations suggest that M. bovis has a particular tropism and pathogenicity for the URT, mainly in the nasal cavity, trachea, and primary bronchi, as previously described (29–32). This is correlated with the high bacterial loads found in the nasal cavities and the trachea. However, we were not able to reproduce typical clinical signs of severe bronchopneumonia in the M. bovis-infected calves, nor to observe macroscopic and microscopic lesions at 6 or 21 dpi in lung tissues. Furthermore, the M. bovis DNA loads detected in the BAL fluid samples at 2 and 7 dpi were low, which could explain the absence of gene transcriptional activation in BAL fluid samples at 2 dpi and the overexpression at 7 dpi of only a few genes (*TLR2*, *TLR4*, *IL-1α*, *IL-1β*, *IL-8*, *TNF-α*, and *CCL3)*, contrary to what was detected in calves infected with IDV or IDV+M. bovis. Altogether, these data could suggest an effective and rapid pulmonary bacterial clearance and/or a limited tropism and/or pathogenicity of M. bovis strain RM16 to the lung parenchyma under our experimental conditions. On the other hand, since the clinical signs were mainly observed from 8 to 18 dpi, it is possible that we sampled tissues too early and missed lesions that would have correlated with the clinical peak at 13 dpi. We observed histopathological lung lesions in one M. bovis-infected calf at 21 dpi, as observed in other publications when calves were necropsied at 21 to 60 dpi (29–32). Further kinetic analyses will be necessary to properly characterize the role of M. bovis strain RM16 in BRD of calves.

The main result here is the fact that, when associated, IDV and M. bovis enhanced respiratory clinical signs and shortened the incubation period. Compared to the clinical signs of the M. bovis-infected calves, those of the coinfected animals were similar yet more severe, suggesting that IDV facilitates M. bovis disease. We also detected more respiratory signs of bronchopneumonia in the coinfected calves than in the IDV-infected calves, suggesting that each pathogen may potentiate the clinical effect of the other. The enhanced disease during coinfection did not seem to be statistically correlated with differences of IDV and M. bovis replication in nasal secretions, despite slightly higher bacterial loads in nasal swab samples of the coinfected group between 2 and 10 dpi (statistically different at 4 dpi). On the other hand, the genomic DNA loads of M. bovis were significantly higher in BAL fluid samples at 7 dpi and in the trachea at 6 dpi when calves were coinfected with IDV, suggesting that IDV facilitates the replication of M. bovis in the LRT during the first days of infection. This may explain the macroscopic and histologic observations showing that the coinfection promotes not only tracheobronchitis but also interstitial bronchopneumonia with fibrinopurulent exudate and lung atelectasis. This also correlates with the observation of more severe clinical signs of LRT infection between 3 and 8 dpi for the coinfected calves than for monoinfected calves. Two studies of coinfections in calves were published using IDV or M. bovis, but they were performed in a sequential mode, with a viral infection first, followed by a bacterial infection. The first study showed that bovine herpesvirus type 1 facilitates the pathology induced by M. bovis in cattle, including chronic pneumonia and polyarthritis syndrome in calves (27). In the second study, Zhang et al. failed to exacerbate the respiratory signs in calves when they performed an IDV infection followed 5 days later by an M. haemolytica infection (26). Several factors, such as the IDV strain, different bacterial species, or coinfection versus temporally separated infection may explain differences in our study. In the study of Zhang et al., the low pathogenic challenge with M. haemolytica, as observed in monoinfected calves, may also explain the absence of enhanced symptomatology in coinfected animals (26).

When we analyzed the transcriptional response of white cell genes in BAL fluid samples at 2 dpi, both IDV and IDV+M. bovis infections had induced rapid differential expression of a high number of genes (37/52 and 32/52 genes, respectively) compared to the gene expression in the control calves. These genes are involved in the innate immune response and especially in the interferon type I signaling and the proinflammatory chemokine and cytokine responses. The overexpression of *TNF-α*, *IL-1α*, *IL-1β*, *CCL2*, *CCL4*, *IL-8*, and *CX3CL1* mRNAs from BAL fluid samples of IDV-infected calves at 2 and 7 dpi correlates with the increases of the total leukocyte counts in the BAL fluid samples at 7 and 14 dpi, partly due to the increase of macrophages, monocytes, and neutrophils. At 7 dpi, the high levels of lymphocytes and *ifn-γ* transcription suggest macrophage and dendritic cell activation, as highlighted by the overexpression of proinflammatory *IL-1α* and *IL-1β* cytokines during IDV infection. At the same time, IDV infection also induced neutrophil diapedesis in the lungs, as determined by the high white cell counts in BAL fluid samples and corresponding to the overexpression of *CCL3*, *CXCL2*, and *IL-8* genes. In the mouse model, primary influenza A virus (IAV) infection was shown to increase macrophages and neutrophils in the airway lumen but was associated with dysfunctions (like phagocytosis) that prevented bacterial clearance and promoted a secondary bacterial infection (33). Conversely, IAV infection was also shown to induce alveolar macrophage depletion, increasing the susceptibility to secondary infection (21, 34, 35). In agreement with our cattle study, Skelton et al. (36) recently showed that intratracheal IDV infection in mice increased the recruitment of neutrophils and lymphocytes and did not deplete macrophage counts in the lungs of mice euthanized at 7 dpi. On the other hand, no clinical signs were observed in another study after IDV infection alone in mice (28). This contrasts with the natural model of the calf where respiratory clinical signs were clearly described (17, 18).

Although not statistically different, coinfection with IDV and M. bovis induced a global RNA expression pattern with higher fold changes than IDV alone that suggested that it favors the transcription of genes involved in the innate and adaptive immune responses. At 7 dpi, 22 genes in the coinfected calves were statistically overexpressed in comparison with their expression in the control animals, which was not the case for the IDV-infected calves. This coincides with higher replication of M. bovis in the BAL fluid samples and lung histopathological lesions in the coinfected group, suggesting that the extended innate immune response against IDV may participate in the severity of M. bovis pathogenicity. Surprisingly, and despite chemokine mRNA overexpression in the coinfected group (especially for *CCL2*, *CCL8*, *ISG15*, and *IFN-γ* genes), the monocyte/macrophage rate increased only slightly at 7 dpi. At 14 dpi, there even seemed to be a depletion of monocytes/macrophages in the BAL fluid samples in this group. We do not know if the presence of M. bovis may limit the recruitment or may contribute to the destruction of macrophages in the airway lumen, thus facilitating the severity of the disease. In addition, even if all three types of infections induced neutrophil diapedesis in calves, the neutrophil fraction in the coinfected animals at 2 and 7 dpi (23.4 and 38.3%, respectively) played an important contribution in increasing the number of leukocytes in the BAL fluid samples. This is consistent with previous studies where IAV superinfection with Staphylococcus aureus or Streptococcus pneumoniae in mice 3 to 6 days later resulted in enhanced neutrophilic inflammation in the lungs (37, 38). On the other hand, similar transcription rates were observed at 2 dpi between calves of the two groups for the *CCL3*, *CXCL2*, and *IL-8* genes (37, 38). We do not know if these earlier and higher numbers of neutrophils in the BAL fluid samples of the IDV+M. bovis-infected calves at 2 dpi contribute to the rapid host response or facilitate the emergence of clinical signs. As we performed coinfections and not superinfections, comparisons with previous publications of IDV superinfections in calves or mice are difficult. Using IDV, Skelton et al. showed that intratracheal IDV inoculation in mice protected against a secondary Staphylococcus aureus infection (36), suggesting that macrophages may be involved in mediating protection from secondary bacterial challenge by neutrophils and lymphocytes. In addition to differences in infection kinetics, the types of bacteria used in each challenge may also explain the exacerbation of the respiratory disease we observed for IDV coinfection with M. bovis in calves. In the mouse model, Skelton et al. suggested that the IFN-β response after IDV infection prevented susceptibility to secondary bacterial infection (36). Conversely, in the IAV*-*S. aureus superinfection model, influenza virus-induced type I IFNs have been shown to inhibit IL-23 production, which is required for the generation of type 17 immunity and for effective clearance of secondary S. aureus pneumonia (37). In calves, our results support that IDV alone or in association with M. bovis induced at 2 and 7 dpi a similar overexpression of ISG mRNAs (mainly *MX1*, *PKR*, *IFIH1*, *IFITM1*, *IFITM3*, and *ISG15*), confirming the activation of the IFN-I pathway by the virus but without statistical differences between the two groups. Consequently, the overexpression of IFN-I-related genes probably does not explain disease differences.

Finally, when we performed direct comparisons between infected groups, almost the same genes were differentially expressed at 2 dpi when M. bovis infection was compared to IDV or IDV+M. bovis infections. At 7 dpi, differences were more numerous between the M. bovis and IDV+M. bovis groups, suggesting a cooperative effect of these two pathogens. When the coinfected group was compared to the two monoinfected groups at 7 dpi, only three genes were statistically differentially expressed: the *IFN-γ* gene with relative overexpression and *PCSK-6* and *TMPRSS2* with relatively lower expression in coinfected animals. In particular, the *IFN-γ* gene was shown to be the most statistically overexpressed gene very quickly after infection at 2 dpi (FC of 31.7) and at least until 7 dpi (FC of 13.2). This was confirmed by higher production of IFN-γ protein in the coinfected group than in the other groups. This correlates with the high level of lymphocytes in BAL fluid samples of coinfected calves mainly at 7 dpi. It could be speculated that this cytokine, mainly produced by natural killer (NK) cells, T helper 1 (Th1) cells, and cytotoxic T lymphocytes (CTL), is directly involved in the enhanced disease of coinfected animals. Indeed, previous publications have reported that mice deficient in IFN-γ-mediated signaling were protected from IAV-S. aureus superinfection (37) and that depletion or dysfunction of alveolar macrophages during IAV infection in mice depends on the level of IFN-γ production and the mouse strain (33, 39), confirming the importance of this cytokine in bacterial phagocytosis by the macrophages.

As mainly observed in BAL fluid samples and serum samples but also in nasal swab samples, the IgA levels were higher in coinfected than in IDV-infected calves at 14 and 21 dpi. This piece of information correlates with the increase of lymphocyte counts in BAL fluid samples from 7 dpi in this group. As previously described for other viruses (40–42), we can postulate that the elevated IgA levels in coinfected calves may contribute to the earlier reduction of IDV loads in the respiratory tract of these animals.

Dysregulations of other genes known to play a role in influenza pathogenesis were detected. *PCSK-6* and *TMPRSS2* encode serine endoproteases (PACE4 and TMPRSS2) that process proproteins trafficking through regulated or constitutive branches of the secretory pathway. While the role of PACE4 in the proteolytic activation of the hemagglutinin of IAV is still being discussed, the TMPRSS2 protein was recently shown to be essential for the spread and pathogenesis of H1N1, H3N2, and H7N9 IAV strains, to facilitate the entry into host cells by proteolytic cleavage and activation of the hemagglutinin (HA) protein (43–45). This protein is highly expressed in the respiratory tract, especially in the lungs (46). Our study clearly shows that the transcription of these two genes is repressed at 7 dpi in the coinfected calves. Interestingly, the *furin* gene also seemed underexpressed when coinfection was compared to IDV monoinfection at this time point. The reason why these genes, which are known to facilitate influenza virus spread, are underexpressed in the coinfected group remains to be elucidated, but their downregulation at 2 and 7 dpi precedes the decrease of IDV RNA loads in BAL fluid samples and lung tissues at 14 and 21 dpi, respectively. This piece of information confirms the importance of exploring the capacity of these proteases to activate the IDV hemagglutinin-esterase-fusion (HEF) glycoprotein, as suggested by Su et al. (47).

To conclude, the results of the present study show that IDV and M. bovis coinfection in calves is associated with extension of the distribution of M. bovis in the lung, exacerbated respiratory pathogenicity, and a strong and prolonged transcriptomic innate immune response in the LRT, especially highlighted by IFN-γ overexpression. Further studies are being carried out to decipher the cellular mechanisms involved.

## MATERIALS AND METHODS

### Virus and bacteria.

The influenza D virus strain D/bovine/France/5920/2014 was isolated from the lung of a dead calf with clinical signs of BRD (9). The virus was propagated on human rectal tumor cells (ATCC CRL-11663) in Dulbecco’s modified Eagle’s medium (DMEM; Dutscher, France) supplemented with 1 μg/ml TPCK (tosylsulfonyl phenylalanyl chloromethyl ketone)-trypsin (Thermo Fisher Scientific, MA, USA) at 37°C, 5% CO_2_ for 5 days. Viral titer was determined on swine testis cells (ATCC CRL-1746) using the 50% tissue culture infective dose (TCID_50_) method as previously described (17).

M. bovis strain RM16 was isolated in 2016 in France from a pool of transtracheal aspiration samples from 3 heifers with clinical signs of respiratory infection (48). Mycoplasma cells were grown in SP4 medium (49) supplemented with cephalexin (500 μg/ml). After 24 to 36 h of incubation at 37°C, mycoplasma cultures were stored at −80°C. Mycoplasma titers were determined by serial dilutions in Dulbecco’s phosphate-buffered saline (PBS; Invitrogen) supplemented with 1% heat-inactivated horse serum (Invitrogen). Dilutions were spotted (10 μl) onto solid SP4 medium, and CFU were counted after 2 to 5 days of incubation at 37°C. For animal inoculations, mycoplasma cells were washed twice in DMEM by 20 min of centrifugation at 9,000 × *g* and kept on ice. Prior to inoculation, 10^10^ CFU were diluted in 10 ml DMEM.

### Safety and ethics.

The animal experiment was performed in biosafety level 3 facilities at the Research Platform for Infectious Disease (PFIE, National Institute for Agronomic Research, INRAE, Nouzilly, France) in accordance with humane standards of animal care (50) and under a national ethical agreement (number APAFIS 16364-2018080211232403; French Ministry of Agriculture, Ethics Committee no. 019).

### Calves and experimental infections.

Twenty-nine Normand and Holstein calves, born at the experimental farm of INRAE-Le Pin (Exmes, France), were used in this study. At birth, the calves received anti-IDV- and anti-M. bovis-specific-antibody-free colostrum (SLU, Sweden). They were transferred to the PFIE at the age of 3 to 6 days. The animals were fed twice a day with commercial reconstituted milk and pellets during 3 to 8 weeks before inoculation. Before challenge, all calves were confirmed to be negative for the presence of M. haemolytica, P. multocida, M. bovis, H. somni, BCoV, IDV, BRSV, and BPIV-3 pathogens in nasal swab samples by real-time PCR (Bio-T respiratory qPCR kits; BioSellal, France). The absence of BVDV was confirmed by analyzing the nonstructural protein 3 (NS3) antigen by ELISA in serum (SERELISA BVDV-BD; Synbiotics, Lyon, France). Before the challenge, calves were confirmed negative for M. bovis- and IDV-specific antibodies by ELISA (Bio K 302; BioX diagnostics, Belgium) and hemagglutination inhibition (HI) assay, respectively. The 29 calves were split into the following four groups (the age distribution was harmonized) in separate rooms: control (uninfected) (*n* = 5), IDV infected (*n* = 8), M. bovis infected (*n* = 8) and IDV+M. bovis infected (*n* = 8) (Fig. 1). Infected calves were inoculated at day zero by nebulization as previously described with 10^7^ TCID_50_ of influenza virus D/bovine/France/5920/2014 and/or 10^10^ CFU of M. bovis RM16 in 10 ml of DMEM (18, 30). Control animals were inoculated with 10 ml of DMEM.

### Clinical observation.

Calves were examined by the same veterinarian twice a day from 3 days before infection to 21 dpi for their general state, decreased appetite during feeding, rectal temperature, nasal discharge, coughing, abnormal breathing, respiratory rate, and abnormal lung sounds. Clinical scores were assessed for each calf as previously described (3) with slight modifications. Briefly, scores for rectal temperatures (*T*) were 0 (*T* < 39°C), 1 (39.1°C < *T* < 40°C), 2 (40.1°C < *T* < 41°C), or 3 (*T* > 41°C); scores for respiratory rates per minute (RR/min) were 0 (RR < 35), 1 (35 < RR < 40), 2 (41 < RR < 60), 3 (61 < RR < 80), or 4 (RR > 80); scores of 0 (normal), 1 (mild), or 2 (severe) were given for nasal discharge and general state; and dyspnea (absent, weak, moderate, or high) and appetite (drop in milk consumption of 0%, <30%, 30 to 60%, or > 60%) were scored from 0 to 3. Finally, two scores were assigned for coughing according to frequency (0, absent; 1, occasional cough; or 2, frequent cough) and severity (0, absent; 1, moderate; or 2, strong cough with violent efforts). Daily individual accumulated scores were calculated for each calf by adding the score of each parameter. Finally, the mean ACS was calculated for each group as the mean of the individual areas under daily clinical scores using the trapezoid method (GraphPad, La Jolla, CA USA).

### Gross lesions and histopathology.

Three animals per infected group (randomly selected before the challenge) were euthanized at 6 dpi to assess the early lesions. The remaining calves (five per group) were euthanized at 21 dpi. Euthanasia was carried out by intravenous injection of pentobarbital sodium (Dolethal, 180 mg per kg of body weight; Vetoquinol, France) followed by complete exsanguination. The tissue samples collected were as follows: right and left cranial, middle, and right and left caudal lobes of the lungs, nasal and tracheal mucosa, tonsils, lymph nodes (mediastinal, tracheobronchial, and mesenteric), olfactory bulb, kidney, spleen, liver, and intestine (duodenum, jejunum, ileum, and colon). Every tissue and organ sample was divided into three parts, one in 10% buffered formalin for histology and two stored at −80°C for RNA and DNA extractions. Examination and scoring of gross lesions, tissue sampling, and histopathology were carried out as previously described (18). For histopathology, a pathologist described and scored the severity of microscopic lesions in slides of lung and respiratory lymph node samples as either light (3 or fewer small lesion foci in one section, score of 1), moderate (>3 small lesion foci per section, score of 2), or marked (diffuse lesions in the section, score of 3).

### Sample collection and preparation.

Nasal swabs were collected daily from 3 days before infection to 21 dpi in 1 ml of PBS and stored at −80°C until extraction of nucleic acids. To quantify mucosal IDV-specific IgG1 and IgG2, sanitary tampons were introduced every 4 days into the left nasal cavity of each calf for 10 min during milk meals. The tampons were removed and pressed to obtain mucosal fluids (between 2 to 5 ml), which were stored at −80°C until ELISAs could be performed. Five days before infection and 2, 7, and 14 dpi, BAL fluid samples were obtained from the same 5 calves per group. Briefly, local anesthesia of the nasal cavities was carried out using a nasal spray containing 2% xylocaine. Five minutes later, the calf was restrained and a first sterile tube (food-grade silicone, 35 cm long, with external and internal diameters of 12 and 8 mm, respectively) covered with anesthetic ointment (2% xylocaine) at the end was introduced into the right nasal cavity and then into the proximal trachea. A second tube (food-grade silicone [Vitryl], 130 cm long, with external and internal diameters of 6 and 4 mm, respectively) was introduced into the first tube and pushed over 1 meter deep until meeting resistance inside the narrow bronchial lumen. Quickly, 100 ml of sterile isotonic sodium chloride solution was injected down the BAL tube using a sterile syringe and immediately aspirated. Between 50 and 80 ml was recovered. The supernatants and white cells from BAL fluid samples (obtained after centrifugation of 30 ml at 300 × *g* for 20 min) were stored at −80°C. Fecal swab samples were collected the day before infection and at 2, 5, 8, 11, and 14 dpi in 2 ml of PBS and stored at −80°C. Blood samples were collected 1 day before infection and at 3, 7, 10, 14, and 21 dpi. Serum was extracted from the blood by centrifugation (800 × *g* for 20 min) and was stored at −20°C.

### BAL fluid samples and white cell populations.

The cellular composition in BAL fluid samples was determined using the Cytospin 3 system (Thermo Fisher Scientific, MA, USA) and MGG staining. Briefly, 10 ml of fresh BAL fluid was centrifuged at 300 × *g* for 20 min. Cell pellets were resuspended in 500 μl of PBS. The Cytofunnels mounted with slides were loaded with 125-μl amounts of resuspended cells. After centrifugation at 300 × *g* for 10 min, the slides were stained with May-Grünwald and Giemsa solutions (RAL Diagnostic, France). A minimum of 100 cells was observed under an optical microscope to identify the white cell populations in BAL fluid samples.

### RNA and DNA extractions.

Viral RNA and bacterial DNA were extracted from 170-μl amounts of nasal swab, raw BAL fluid, and fecal swab samples using the NucleoMag pathogen kit (Macherey-Nagel, Germany) on the KingFisher Flex purification system (Thermo Fisher Scientific, MA, USA) according to the manufacturer’s instructions. Total RNA was extracted from pelleted cells of BAL fluid samples with the NucleoMag RNA kit (Macherey-Nagel, Germany) on the KingFisher Flex purification system (Thermo Fisher Scientific, MA, USA). Extraction of viral RNA and bacterial DNA from tissues (30-mg amounts of nasal turbinate, trachea, and lung lobe samples) were obtained by lysis in Precellys lysing kit tubes (catalog number P000912-LYSKO-A; Bertin Technologies, France) with 500 μl of Opti-MEM on a Precellys system (Bertin Technologies, France). Nucleic acids were then extracted using the NucleoSpin RNA virus kit (Macherey-Nagel, Germany).

### IDV and M. bovis quantification.

Influenza D virus was quantified in nasal swab, BAL fluid, and tissue samples using a one-step RT-qPCR as previously described (10). Briefly, the viral polymerase basic 1 (PB1) gene was amplified with specific primers and quantified by using a specific probe and the QuantiNova probe RT-PCR kit (Qiagen, Germany) on a LightCycler 96 real-time PCR system (Roche, Switzerland). Viral copy numbers in samples were determined by using a standard plasmid containing the PB1 product of influenza virus D/bovine/France/5920/2014 (18). For quantification of M. bovis genomic DNA copy numbers in nasal swab, BAL fluid, and tissue samples, qPCR was performed with specific primers, probe, and specific standard using the Bio-T Mycoplasma bovis PCR kit (Biosellal, France) on the LightCycler 96 real-time PCR system (Roche, Switzerland) according to the manufacturer’s instructions.

### Host humoral response.

The IDV-specific antibody titers in animal sera were determined by HI assay as previously described (18). The IDV-specific IgG1 and IgA raised against IDV in BAL fluid, nasal swab, and serum samples were assessed using either indirect or capture ELISAs. The indirect ELISA was as previously described (18, 51), and the capture ELISA was designed according to Uttenthal et al. (52). ELISA plates (MaxiSorp; Nunc, Denmark) were coated for 18 h at 4°C with 100 ng mouse anti-bovine IgA (interleukin A71 [IL-A71], catalog number MCA2438; Bio-Rad Laboratories, Inc., Solna, Sweden) per well in coating buffer containing 0.05 M Na-carbonate bicarbonate, pH 9.6. The wells were thereafter blocked for 1 h at 25°C with PBS containing 2% (wt/vol) bovine serum albumin, before the addition of (i) samples diluted 1:25 (nasal secretions) or 1:10 (BAL fluid), (ii) IDV antigen or cell control (cell culture medium from CRL1756 cells infected with influenza virus D/bovine/France/5920/2014 or similar uninfected cells), (iii) mouse anti-IDV antibodies (monoclonal antibody [MAb] 4F1) produced and characterized as described previously (31), or (iv) rat anti-mouse IgG1 conjugated with horseradish peroxidase (HRP) (clone LO-MG1-2, catalog number MCA336P; Bio-Rad), TMB (3,3′,5,5′-tetramethylbenzidine) substrate, and H_2_O_2_. Each antibody or antigen solution was added in a volume of 100 μl per well and incubated for 1 h at 37°C, prior to three washes with 0.05% PBS–Tween 20 solution. To analyze the IDV-specific IgG1 and IgA titers, the optical density (OD) was measured at 450 nm on a microplate reader. Corrected OD (COD) values were calculated by subtracting the OD values of wells containing control antigen from those of wells containing IDV antigen. Data were expressed as percent positivity (PP) values, corresponding to the COD of the sample divided by the COD of the positive control. Detection of antibodies against M. bovis was performed with a commercial ID Screen Mycoplasma bovis indirect ELISA kit (Innovative Diagnostics, France). The antibody titers were calculated according to the manufacturer’s guidelines.

### Transcriptomic analyses from BAL fluid samples.

Host gene expression levels in the BAL fluid samples collected 5 days before infection and at 2, 7, and 14 dpi were compared. Briefly, cDNA synthesis was performed from 100 ng of RNA from BAL fluid samples by using random hexamer primers and RevertAid reverse transcriptase (Thermo Fisher Scientific, MA, USA) according to the manufacturer’s instructions. The sets of primers were previously selected (17), and new primer pairs were designed from bovine RNA sequences. Gene primers were preferentially designed between two exons by using Primer3 software and validated by using an RT-qPCR protocol on the LightCycler 96 real-time PCR system (Roche, Switzerland). The primer pairs and targeted genes are listed in Table S1. A total of 76 bovine genes playing roles in the antiviral response, inflammatory response, chemotaxis, and/or immune cell differentiation and 8 housekeeping genes were studied as described previously (18). The quantifications were performed using the Biomark microfluidic system from Fluidigm (GeT-PlaGe platform, France), in which every sample-gene combination is quantified using a 96.96 Dynamic Array IFC (integrated fluidic circuit) (product number bBMK-M-96.96; Fluidigm). Specific target amplification (STA) was performed on the 80 cDNA samples, an internal control (genomic DNA), a negative control (Tris-EDTA [TE]), a bovine genomic DNA control, and pooled cDNAs of the 80 cDNA samples in 5-fold serial dilutions (to determine the PCR amplification efficiency). Each sample was preamplified with a pool of selected primer pairs (0.2 μM each primer) in a thermocycler with an initial activation step (95°C for 10 min) followed by 16 cycles of two amplification steps (95°C for 15 s and 60°C for 4 min). The free primers present in the preamplification products were digested with an exonuclease (NEB, MA, USA) before the samples were diluted in TE with a 1:5 dilution factor. The preamplification, digested, and diluted products were prepared with 50% 2× TaqMan gene expression master mix (Applied Biosystems, CA, USA), 5% 20× DNA binding dye sample loading reagent (Fluidigm, CA, USA), 5% EvaGreen (Interchim, France), and 15% 1× TE in a 10-μl final volume. The BioMark 96.96 Dynamic Array chip (Fluidigm, CA, USA) was loaded with 5 μl of each prepared sample. Gene expression levels were measured after 35 cycles on the BioMark HD real-time PCR system. The quality control was checked by using the Fluidigm software (Fluidigm, CA, USA).

A clean data set was obtained after analysis of the melting curves using Fluidigm real-time PCR analysis software version 4.1.3. As a standard, a pool of the 80 cDNA samples was used in five serial dilutions to determine the gene amplification efficiencies (Eff). Genes with amplification efficiencies lower than 1.7 or higher than 2.2 and genes with fewer than three of the five standard points amplified were removed from the analysis.

The relative gene expression (RE) for each sample was calculated as proposed by Pfaffl (53) with the equation RE_(gene)_ = Eff_(gene)_^^(^*^CT^* ^calibrator −^ *^CT^* ^sample)^, where *CT* is cycle threshold and the calibrator is the first point of the standard curve (Table S2). GeNorm was used to choose the four most stable housekeeping genes. The select HKgenes function with the Vandesompele method (54) of the SLqPCR package was used with RStudio (version 1.2.1335). Finally, the relative expression level for each sample was normalized for the geometric average expression of the selected housekeeping genes as follows:

normalized RE(gene)=RE(gene)/(RE(RPL19)×RE(RPL26)×RE(SOD2)×RE(YWHA7))3

A cutoff was chosen to only select genes with less than 40% missing values. In total, 52 of 76 genes were retained for this study. The normalized relative expression of these 52 genes was log transformed, and missing values were imputed using the missMDA package of R software.

### Bovine IFN-γ quantification.

The concentrations of bovine IFN-γ in BAL fluid samples were measured by using a homemade sandwich ELISA. The mouse anti-bovine IFN-γ antibody (clone CC330, catalog number MCA2112; Bio-Rad) was incubated on ELISA plates (MaxiSorp; Nunc, Denmark) for 18 h at 4°C using 100 ng per well in 100 μl coating buffer containing 0.05 M Na-carbonate bicarbonate, pH 9.6. After three washes with 100 μl of wash buffer (PBS containing 0.05% Tween 20), wells were blocked for 1 h at room temperature with 100 μl of PBS containing 1% bovine serum albumin (BSA). After three washes, 100 μl of 1:2-diluted BAL fluid samples were added. A standard range was done using the recombinant bovine IFN-γ (catalog number PBP007A; Bio-Rad) diluted in wash buffer at a final concentration between 0.0125 and 125 ng/ml. After incubation overnight at 4°C and three washes, 100 μl of biotinylated mouse anti-bovine IFN-γ antibody (clone CC302, catalog number MCA1783B; Bio-Rad) labeled with streptavidin-HRP was added to each well. After incubation at 37°C for 1 h, the ELISA plates were washed three times. Absorbance was read at 450 nm on the CLARIOstar microplate reader (BMG Labtech) after the addition of 100 μl HRP substrate (Bio-Rad).

### Statistical analysis.

Statistical analysis for clinical, serological, and virological analyses was performed using GraphPad (La Jolla, CA, USA). Logarithmic transformation was applied to fulfill the conditions of variances in homogeneity and normality when necessary (qPCR data). Data were expressed as arithmetic mean values ± standard errors of the means (SEM). A two-way analysis of variance (ANOVA) with repeated measures (3-factor split-plot ANOVA) was used to analyze the clinical, ELISA, and qPCR results. When effects of the “day” and “treatment” factors were significant among interactions, a Bonferroni test between contrasts was used to compare the treatments on each day postchallenge. A one-way ANOVA was used to compare the means of the individual areas under daily clinical scores (ACS). When the effect of the “treatment” factor was significant, a Newman-Keuls test was used to compare the treatment effects at each time point. A *t* test (Mann-Whitney test) was also run for these parameters.

Statistical analyses of the DEGs between samples at the different stages of infection (2, 7, and 14 dpi) were obtained using R Studio software. Data for each sample were analyzed with a linear model that used the animal as a random parameter and the type of infection as a fixed parameter. Correction for multiple testing was performed with the estimation of false discovery rate as proposed by Benjamini and Hochberg (55). After correction, differentially expressed genes with a significant difference (*P* < 0.05) were identified. Finally, for each comparison, the fold change was calculated and represented.
